# A Non-Nucleotide STING Agonist MSA-2 Synergized with Manganese in Enhancing STING Activation to Elicit Potent Anti-RNA Virus Activity in the Cells

**DOI:** 10.3390/v15112138

**Published:** 2023-10-24

**Authors:** Hanrui Lin, Rui Zhang, Hanyi Xiang, Xinqian Lin, Xiongting Huang, Jingsong Chen, Long Zhou, Zhidong Zhang, Yanmin Li

**Affiliations:** Key Laboratory of Animal Medicine of Sichuan Province, College of Animal and Veterinary Sciences, Southwest Minzu University, Chengdu 610093, China

**Keywords:** MSA-2, manganese, stimulator of interferon genes, Seneca Valley virus, antiviral effect

## Abstract

Both Manganese (Mn^2+^) and MSA-2 can activate the downstream signal pathway through stimulator of interferon genes (STING) and induce the expression of type I interferon, which is important for hosts to protect against DNA viruses. However, its effect on RNA viruses remains unknown. In this study, we used Seneca Valley virus (SVV) as a model RNA virus to investigate the inhibitory effects of Mn^2+^ and MSA-2 on the virus replication in the porcine cells (PK-15 cells). The results showed that both MSA-2 and Mn^2+^ were able to inhibit the SVV replication in PK-15 cells. The combination of MAS-2 and Mn^2+^ could confer better protection against SVV. Further studies showed that MSA-2 and Mn^2+^ could activate TBK1, IRF3 and NFκB through STING and induce the expression of IFN-β, IL-6 and TNF-α. The present study confirmed that MSA-2 synergized with Mn^2+^ in STING activation to generate a better antiviral effect in vitro, which would be helpful for the further development of effective antiviral drugs in the future.

## 1. Introduction

Stimulator of interferon genes (STING) localized at the ER membrane plays a key role in regulating innate immune responses to viral infection [1]. Activating STING recruits TANK-binding kinase-1 (TBK1) and then promotes the phosphorylation of TBK1; subsequently, it recruits and phosphorylates interferon (IFN) regulatory factor 3 (IRF3), leading to the initiation of antiviral effects [1]. Therefore, recent studies have shown that use of the STING ligand to activate innate immunity is a promising approach to protect against viral infection [2,3].

Non-cyclic dinucleotide (CDN) STING ligand MSA-2 is a non-nucleotide human and mouse STING agonist, which was recently identified as a new STING agonist with systemic in vivo activity [4]. It binds to STING in the form of a non-covalent dimer, which makes STING form a closed conformation, activates downstream signal pathways, and induces the expression of type I interferon [4]. A study has shown that MSA-2 has good anti-tumor immunity via inducing the phosphorylation of TBK1/IRF3 to promote IFN secretion [4]. In addition, it was found that the combination of MSA-2 and anti-PD-1 therapy outperformed monotherapy [5]. However, it remains unknown whether MSA-2 can activate STING in other hosts except human and mice and then induce its antiviral effect.

Manganese (Mn^2+^) is one of the most abundant metals in mammalian tissues, which is necessary for a variety of physiological processes including immune regulation and antioxidant defense [6,7]. There’s a study that demonstrated that Mn^2+^ can promote STING activity by enhancing the binding affinity of 2′, 3′-cyclic guanosine monophosphate-adenosine monophosphate (cGAMP) to STING and then induce the potent immunity against DNA viruses [8]. In addition, it was reported that Mn^2+^ can also induce the immune response against the infection with RNA viruses, including Newcastle disease virus (NDV), foot-and-mouth disease virus (FMDV) and Sendai virus (SeV) [8,9,10]. A recent study discovered that Mn^2+^ is also essential in the innate immune sensing of tumors via cGAS-STING [11]. Therefore, we hypothesized that Mn^2+^ could synergize with MAS-2 to enhance the inhibitory effect on RNA virus replication.

Seneca Valley virus (SVV), also known as Seneca virus A (SVA), is a non-enveloped, positive-stranded, single-stranded RNA virus, which belongs to the Senecavirus genus in the family *Picornaviridae* [12]. The virus can cause vesicular disease in pigs mainly characterized by blisters and ulceration in the mouth, nose and hoof crown of the infected pigs, and even death in serious cases, which is clinically similar to foot-and-mouth disease and swine vesicular disease [13] and has caused significant economic losses to the global pig industry [14]. At present, there is no commercial vaccine available.

In the present study, SVV was used as a model virus to analyze the anti-RNA viruses activity of MAS-2 and Mn^2+^ in porcine PK-15 cells and then evaluate if Mn^2+^ could enhance MAS-2 to elicit potent immunity to SVV infection. The results showed that both MAS-2 and Mn^2+^ can induce protection against SVV infection in PK-15 cells, respectively. Importantly, the combination of MAS-2 and Mn^2^ generated a better antiviral effect. Further studies showed that both of them activated TBK1, IRF3 and NFκB through STING and induce the expression of type I interferon and cytokines IL-6 and TNF-α mRNA. The results indicated that the activation of STING by the combination of MAS-2 and Mn^2+^ could confer better protection against RNA viruses.

## 2. Materials and Methods

### 2.1. Reagents and Antibodies

The MnCl_2_(H_2_O)_4_ was purchased from Sigma Aldrich (CAS: 13446-34-9, St. Louis, MO, USA). The MSA-2 was purchased from MedChemExpress (CAS: HY-141514, Monmouth Junction, NJ, USA). The rabbit monoclonal antibodies anti-STING (D1V5L; catalog number 50494S) and anti-phospho-STING (Ser366 and E9A9K; catalog number 50907S), anti-NFκB p65 XP (D14E12; catalog number 8242S), anti-phospho-NFκB (Ser536 and 93H1; catalog number 3033S), anti-IRF3 (D614C; catalog number 11904S), anti-phospho-IRF3 (Ser396 and D6O1M; catalog number 29047S), anti-TBK1/NAK XP (D1B4; catalog number 3504S) and anti-phospho-TBK1/NAK XP (Ser172 and D52C2; catalog number 5483S) were from Cell Signaling Technology. The mouse monoclonal antibody anti-β-tubulin (catalog number 66240-1-Ig) was obtained from Proteintech. The HRP Goat Anti-Rabbit IgG secondary antibody (catalog number 31460) and goat anti-mouse IgG secondary antibody (catalog number 31430) were from Invitrogen.

### 2.2. Cell Culture

The IBRS cells (porcine kidney cells), PK-15 cells (porcine kidney cells) and STING knockout PK-15 cells were preserved in this laboratory. Cells were cultured in Dulbecco’s modified Eagle’s medium (DMEM) supplemented with 10% fetal bovine serum (FBS), 100 μg/mL penicillin, and 100 μg/mL streptomycin as monolayers in cell culture flasks or dishes at 37 °C under 5% CO_2_.

### 2.3. Virus

SVV was preserved in our laboratory. IBRS cells were inoculated with SVV and incubated at 37 °C under 5% CO_2_ for 1 h. The cells were washed to remove the unbound virus and then cultured in fresh DMEM containing 2% FBS for 13 h. When the cells showed cytopathic changes of about 80%, the cells were frozen and thawed three times, after which they were clarified by centrifugation at 8000× *g* for 10 min. Then, the supernatant was taken and stored at −80 °C. The titer was determined by the Reed–Muench method and expressed as 50% tissue culture infectious dose (TCID_50_).

### 2.4. Drug Treatment and Virus Infection

When the fusion degree of PK-15 cells reached about 90%, it was digested with trypsin, 10% nutrient solution was added to make cell suspension, and the cell suspension was inoculated to cell culture flasks or dishes. The corresponding concentration of MSA-2 or Mn^2+^ was added at the same time. Then, 24 h later, PK-15 cells were mock infected or infected with SVV at a multiplicity of infection (MOI) of 10, incubated at 37 °C under 5% CO_2_ for 1 h to wash off unbound virus, and cultured in fresh DMEM containing 2% FBS. The corresponding concentration of MSA-2 or Mn^2+^ was added at the same time. Cells and cell supernatants were collected 24 h after SVV infection.

### 2.5. Cytotoxicity Assay

When the fusion degree of PK-15 cells reached about 90%, 10% nutrient solution was added to make the cell suspension after trypsin digestion. Then, 100 μL of the cell suspension was seeded into a 96-well plate and cultured at 37 °C under 5% CO_2_ for 24 h. Afterwards, different concentrations of MSA-2 or Mn^2+^ were added with 8 repeats of each concentration, and a blank control group was set up at the same time. After 24 h, 10 μL CCK-8 solution (absin, abs5003) was added to each well and incubated at 37 °C under 5% CO_2_ for 2 h. The absorbance at 450 nm was determined by PerKinElmer VICTOR Nivo.

### 2.6. RNA Extraction and RT-qPCR

Total RNA was extracted using a Thermo Scientific (Waltham, MA, USA) GeneJET RNA purification kit (K0732) and reverse transcribed using a ReverTra Ace qPCR RT Master Mix with gDNA Remover (TOYOBO 121800). According to the manufacturer’s instructions, Powerup™ SYBR™ Green Master (MixThermpfisher A25742) was used for quantitative real-time PCR. qPCR primers are shown in Table 1. Taking the expression level of GAPDH as the internal reference value, the expression level of mRNA was calculated by 2^−ΔΔCt^.

### 2.7. Western Blotting

The cells were harvested and lysed with RIPA cell lysis buffer containing protease inhibitor PMSF on ice, and the cells were broken by ultrasound at 300 W for 1 min, 16,500× *g* at 4 °C, centrifuged for 10 min, and the supernatant was added to the protein loading buffer. The protein samples were subjected to SDS-PAGE and transferred to a polyvinylidene fluoride (PVDF) membrane. The membrane was blocked with 5% nonfat milk for 4 h at room temperature. Then, it was incubated overnight at 4 °C for specific primary antibody, which was followed by incubation with horseradish peroxidase (HRP)-labeled secondary antibodies for 2 h at room temperature. The results were observed with the Chromogenic solution (Affinity, KF8001) under the BLT GelView6000Plus gel imager.

### 2.8. Statistical Analysis

The data were analyzed using the GraphPad Prism 8 software and expressed as means and standard deviations (SD). A *t*-test or one-way ANOVA was used for statistical analysis. *p* < 0.05 was considered statistically significant.

## 3. Results

### 3.1. MSA-2 Inhibits SVV Replication in the Porcine Cells

**Figure 1:**
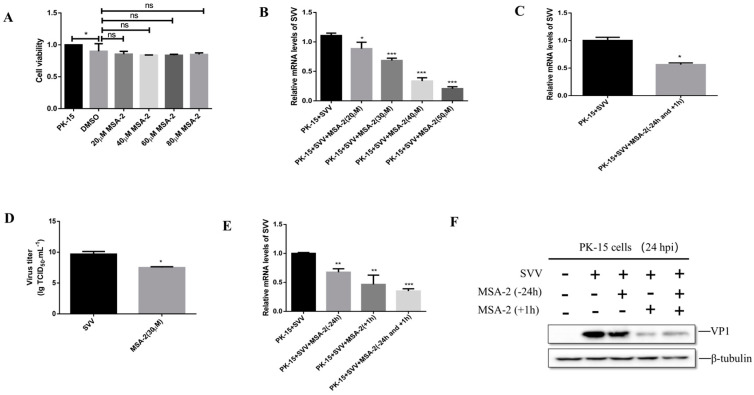
Antiviral activity of MSA-2 against SVV in PK-15 cells. (**A**) PK-15 cells were treated with different concentrations of MSA-2 for 24 h, and the cell viability was detected by the CCK-8 method. (**B**) PK-15 cells were treated with different concentrations of MSA-2, respectively, and infected with SVV. The expression of viral RNA was detected by RT-qPCR. PK-15 cells were treated with 30 μM MSA-2 24 h before infection and 1 h after infection. SVV RNA expression was detected by RT-qPCR (**C**), and the viral titers were detected by TCID_50_ (**D**). PK-15 cells were treated with 30 μM MSA-2 24 h before infection or 1 h after infection or simultaneously before and after infection. SVV RNA expression was detected by RT-qPCR (**E**), and SVV VP1 protein expression was detected by Western blot (**F**). *, *p* < 0.05; **, *p* < 0.01; ***, *p* < 0.001.

To determine the cytotoxicity of MSA-2 to PK-15 cells, the cells were treated with different concentrations of MSA-2 for 24 hours (h), respectively, and the cell viability was then detected by using the CCK-8 method. As shown in Figure 1A, in comparison with the control group, the cell survival rate was greater than 80% when the cells were treated with 20, 40, 60 and 80 μM of MSA-2, respectively. In order to determine the working concentration of MSA-2, the PK-15 cells were treated with MSA-2 at a concentration of 20, 30, 40, and 50 μM, respectively, for 24 h and then infected with SVV at 10 MOI. At 24 h post-infection (hpi), the level of SVV RNA in the treated cells was detected by RT-qPCR. The results showed that compared with the untreated control group, the level of viral RNA was significantly decreased in MSA-2-treated cells in a dose-dependent manner (Figure 1B). Combined with the results of the cytotoxicity test and the inhibitory effect on the virus replication, 30 μM of MSA-2 was selected as the working concentration for further study.

To investigate the antiviral effect of MSA-2 on SVV replication in PK-15 cells, the cells were treated with 30 μM MSA-2 for 24 h before SVV infection and 1 h after infection. At 24 hpi, the viral RNA level (Figure 1C) and virus titer (Figure 1D) were detected by RT-qPCR and TCID_50_, respectively. The results showed that the SVV titer and RNA levels were significantly reduced in the cells treated with MSA-2 when compared with the control group. To further investigate the antiviral effect of MSA-2 on SVV replication, PK-15 cells were treated with 30 μM MSA-2 at 24 h before infection, 1 h after infection, or simultaneously before and after infection, respectively. The results showed that compared with the control group, MSA-2 could inhibit the expression level of viral RNA (Figure 1E) and VP1 protein (Figure 1F) when MSA-2 was used to treat cells, but the inhibitory effect was more significant when MSA-2 was used to treat cells after infection with SVV. These results demonstrated that MSA-2 can inhibit SVV replication in vitro.

### 3.2. MSA-2 Activates STING in the Porcine Cells

**Figure 2:**
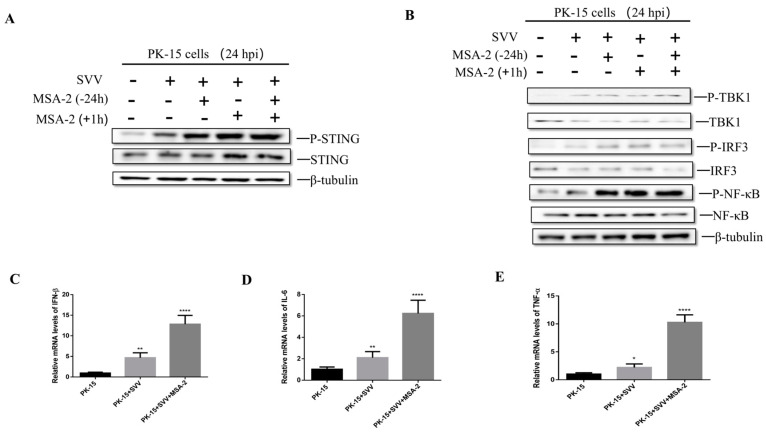
MSA-2 activates innate immunity and induce cytokine expression. PK-15 cells were treated with 30 μM MSA-2 24 h before infection, 1 h after infection, or simultaneously before and after infection. P-STING protein expression was detected by Western blot (**A**), and P-TBK1, P-IRF3, and P-NFκB protein levels were detected by Western blot (**B**). PK-15 cells were treated with 30 μM MSA-2 1 h after infection, and the mRNA levels of IFN-β (**C**), IL-6 (**D**), and TNF-α (**E**) were detected by RT-qPCR 24 h after virus infection. *, *p* < 0.05; **, *p* < 0.01; ****, *p* < 0.0001.

A previous study demonstrated that MSA-2 is a STING agonist in mice and humans [4]. In order to explore whether MSA-2 is able to activate STING in the porcine cells, PK-15 cells were treated with 30 μM MSA-2 for 24 h before infection, 1 h after infection, and simultaneously 24 h before and 1 h after infection, respectively. The activation level of STING and its related proteins was detected. The results showed that P-STING was significantly up-regulated in MSA-2-treated and SVV-infected PK-15 cells when compared with the untreated and infected group (Figure 2A). Subsequently, STING downstream-related proteins were detected, and the results showed that P-TBK1, P-IRF3 and P-NFκB protein levels in MSA-2-treated PK-15 cells infected with SVV were significantly higher than those in the untreated and infected group (Figure 2B). In addition, the PK-15 cells were treated with 30 μM MSA-2 for 1 h after the cells infected with the virus. At 24 hpi, the mRNA levels of IFN-β, IL-6, and TNF-α were detected by RT-qPCR. The results showed that the mRNA levels of IFN-β, IL-6 and TNF-α after MSA-2 treatment were significantly higher than those in the untreated group (Figure 2C–E). These results suggest that MSA-2 can activate the STING signaling pathway and then induce cytokine expression.

### 3.3. MSA-2 Elicits Anti-Virus Effects via Activation of STING

**Figure 3:**
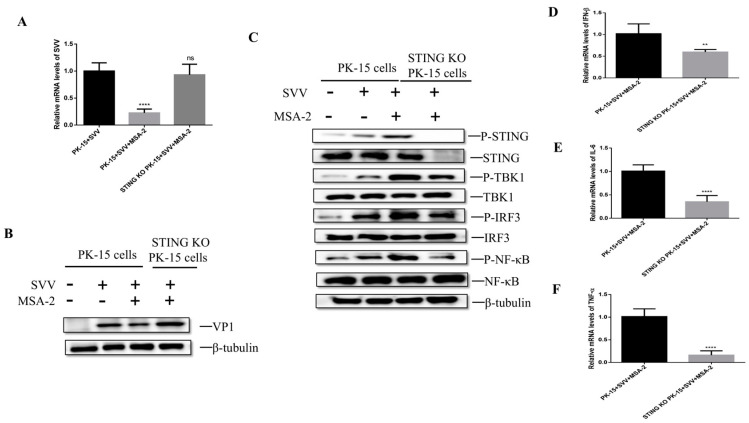
MSA-2 activates innate immunity to resist SVV through STING. PK-15 cells or STING knockout PK-15 cells were treated with 30 μM MSA-2 at 1 h after infection. Viral RNA level (**A**) and SVV VP1 proteins (**B**) were detected by RT-qCPR and Western blot 24 h after infection. (**C**) Cells were treated with 30 μM MSA-2 at 1 h after infection, and P-TBK1, P-IRF3, and P-NFκB protein levels were detected by Western blot at 24 h after infection. Cells were treated with 30 μM MSA-2 at 1 h after infection, and the mRNA levels of IFN-β (**D**), IL-6 (**E**), and TNF-α (**F**) were detected by RT-qPCR at 24 h after virus infection. **, *p* < 0.01; ****, *p* < 0.0001.

In order to explore whether MSA-2 exerts its antiviral effect through STING, STING knockout PK-15 cells were treated with 30 μM MSA-2 at 1 hpi. The viral RNA and VP1 protein levels were detected by RT-qPCR and Western blot. The results showed that MSA-2 failed to inhibit the SVV replication in STING knockout PK-15 cells. When compared with MSA-2-treated and SVV-infected PK-15 cells, the higher levels of SVV RNA (Figure 3A) and VP1 proteins (Figure 3B) were observed in MSA-2-treated and SVV-infected STING knockout PK-15 cells, which is similar to that in the PK-15 cells without MSA-2 treatment. To further explore the antiviral mechanism of MSA-2, the expression of STING-related proteins and the level of cytokine mRNA were detected by Western blot and RT-qPCR, respectively. The results showed that compared with MSA-2-treated and SVV-infected PK-15 cells, the levels of P-TBK1, P-IRF3 and P-NFκB were obviously lower in the STING knockout cells (Figure 3C), indicating that MSA-2 failed to activate TBK1, IRF3 and NFκB in the STING knockout cells. In addition, the IFN-β (Figure 3D), IL-6 (Figure 3E) and TNF-α (Figure 3F) mRNA levels in the STING knockout cells were significantly lower than those in PK-15 cells. These results suggest that MSA-2 exerts antiviral effects through STING activation of innate immunity.

### 3.4. Antiviral Activity of Mn^2+^ against SVV Infection in PK-15 Cells

**Figure 4:**
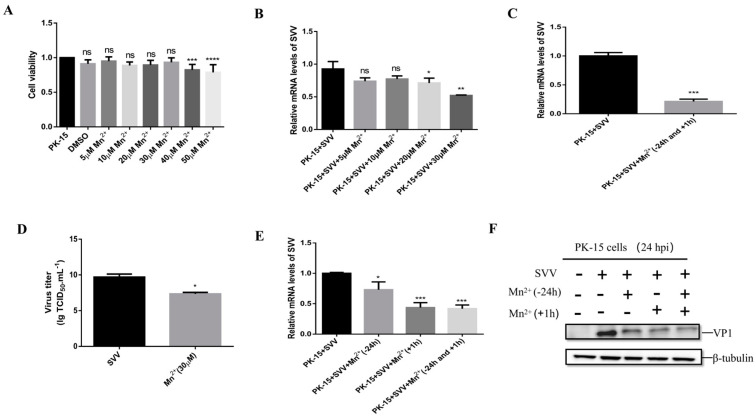
Antiviral activity of Mn^2+^ against SVV in PK15 cells. (**A**) PK-15 cells were treated with different concentrations of Mn^2+^ for 24 h, and the cell viability was detected by the CCK-8 method. (**B**) PK-15 cells were treated with different concentrations of Mn^2+^, respectively, and infected with SVV. The expression of viral RNA was detected by RT-qPCR. PK-15 cells were treated with 30 μM Mn^2+^ 24 h before infection and 1 h after infection. SVV RNA expression was detected by RT-qPCR (**C**), and the viral titers were detected by TCID_50_ (**D**). PK-15 cells were treated with 30 μM Mn^2+^ 24 h before infection, 1 h after infection, or both before and after infection. SVV RNA expression (**E**) was detected by RT-qPCR, and SVV VP1 protein expression (**F**) was detected by Western blot. *, *p* < 0.05; **, *p* < 0.01; ***, *p* < 0.001; ****, *p* < 0.0001.

To determine the cytotoxicity of Mn^2+^ to PK-15, PK-15 cells were treated with different concentrations of Mn^2+^ (5, 10, 20, 30, 40, 50 μM) for 24 h, and the cell viability was detected by the CCK-8 method. The results show that (Figure 4A) compared with the control group, the cell survival rate is greater than 80% when Mn^2+^ at a concentration of 30 μM or less was used to treat the cells, but when the concentration of Mn^2+^ used is 40 μM or 50 μM, the survival rate of the cells is significantly reduced compared with the control group. To determine the working concentration of Mn^2+^, the cells were treated with different concentrations of Mn^2+^ (5, 10, 20 and 30 μM), respectively, for 24 h and then were infected with SVV at 10 MOI. The cells were harvested at 24 hpi, and the level of SVV RNA was detected by RT-qCPR. The results showed that the level of viral RNA in the cells treated with 30 μM Mn^2+^ was significantly lower than that in the untreated cells (Figure 4B). Combined with a cytotoxicity test and RT-qPCR results, 30 μM was finally selected as the working concentration.

To ensure the effect of Mn^2+^ on the replication of SVV in PK-15 cells, the cells were treated with 30 μM Mn^2+^ for 24 h before SVV infected PK-15 cells and 1 h after infection. The viral titer and viral RNA level were detected by TCID_50_ assay and RT-qPCR at 24 hpi. The results showed that compared with the control group, the RNA level (Figure 4C) and viral titer (Figure 4D) were significantly decreased in the cells treated with Mn^2+^. To further investigate the inhibitory effect of Mn^2+^ on SVV replication, the cells were treated with 30 μM Mn^2+^ for 24 h before SVV infection, 1 h after SVV infection or both before and after infection. At 24 hpi, the levels of the viral RNA and VP1 protein were detected by RT-qPCR and Western blot, respectively. The results showed that compared with the control group, whether Mn^2+^ was used to treat the cells before or after infection, the levels of SVV RNA (Figure 4E) and VP1 protein (Figure 4F) were significantly lower, and the inhibitory effect was more significant when cells were treated with Mn^2+^ after the virus infection. These results indicate that Mn^2+^ can inhibit SVV replication in vitro.

### 3.5. Mn^2+^ Activates STING to Inhibit the Viral Replication

**Figure 5:**
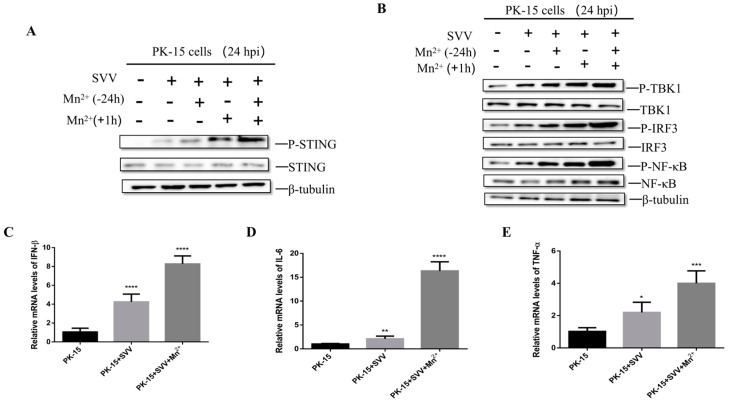
Mn^2+^ can activate innate immunity and induce cytokine expression. PK-15 cells were treated with 30 μM Mn^2+^ 24 h before infection, 1 h after infection, or both before and after infection. P-STING protein expression was detected by Western blot (**A**), and P-TBK1, P-IRF3, and P-NFκB protein levels were detected by Western blot (**B**). PK-15 cells were treated with 30 μM Mn^2+^ 1 h after infection, and the mRNA levels of IFN-β (**C**), IL-6 (**D**), and TNF-α (**E**) were detected by RT-qPCR 24 h after virus infection. *, *p* < 0.05; **, *p* < 0.01; ***, *p* < 0.001; ****, *p* < 0.0001.

In order to explore the antiviral mechanism of Mn^2+^, PK-15 cells were treated with 30 μM Mn^2+^ for 24 h before infection, 1 h after infection, or both before and after infection, respectively. At 24 hpi, the level of STING-related proteins was detected by Western blot. The results showed that in Mn^2+^-treated and SVV-infected cells, the levels of P-STING protein (Figure 5A), P-TBK1, P-IRF3 and P-NFκB (Figure 5B) were significantly higher when compared with the control group. In addition, for the cells treated with 30 μM Mn^2+^ at 1 hpi, the mRNA levels of IFN-β (Figure 5C), IL-6 (Figure 5D), and TNF-α (Figure 5E) were significantly higher than those in the untreated group. These results suggest that Mn^2+^ can also activate the STING signaling pathway and induce cytokine expression.

### 3.6. Mn^2+^ Required STING to Activate Anti-Viral Innate Immunity

**Figure 6:**
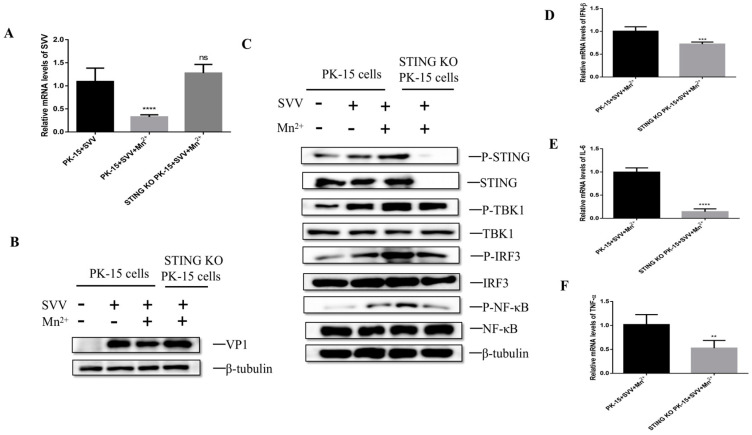
Mn^2+^ activates innate immunity through STING to resist SVV. PK-15 cells or STING knockout PK-15 cells were treated with 30 μM Mn^2+^ 1 h after infection. Viral RNA level (**A**) and SVV VP1 proteins (**B**) were detected by RT-qCPR and Western blot 24 h after infection. (**C**) Cells were treated with 30 μM Mn^2+^ 1 h after infection, and P-TBK1, P-IRF3, and P-NFκB protein levels were detected by Western blot 24 h after infection. Cells were treated with 30 μM Mn^2+^ 1 h after infection, and the mRNA levels of IFN-β (**D**), IL-6 (**E**), and TNF-α (**F**) were detected by RT-qPCR 24 h after virus infection. **, *p* < 0.01; ***, *p* < 0.001; ****, *p* < 0.0001.

To investigate whether the antiviral effect of Mn^2+^ needs the activation of STING, the PK-15 cells or STING knockout PK-15 cells were infected with SVV for 1 h and then were treated with 30 μM Mn^2+^. The levels of viral RNA and VP1 protein were detected by RT-qPCR and Western blot. The results showed that compared with Mn^2+^-treated and SVV-infected PK-15 cells, the levels of the viral RNA (Figure 6A) and VP1 protein (Figure 6B) were increased in STING knockout cells. Further analysis showed that the levels of P-TBK1, P-IRF3, and P-NFκB significantly decreased in the STING knockout cells when compared with Mn^2+^-treated and SVV-infected PK-15 cells (Figure 6C). Moreover, the levels of IFN-β, IL-6 and TNF-α mRNA were also down-regulated in these STING knockout cells (Figure 6D–F). These results indicate that STING is necessary for Mn^2+^ to induce the antiviral effect.

### 3.7. MSA-2 Combined with Mn^2+^ Enhanced Inhibitory Effect on SVV Replication

**Figure 7:**
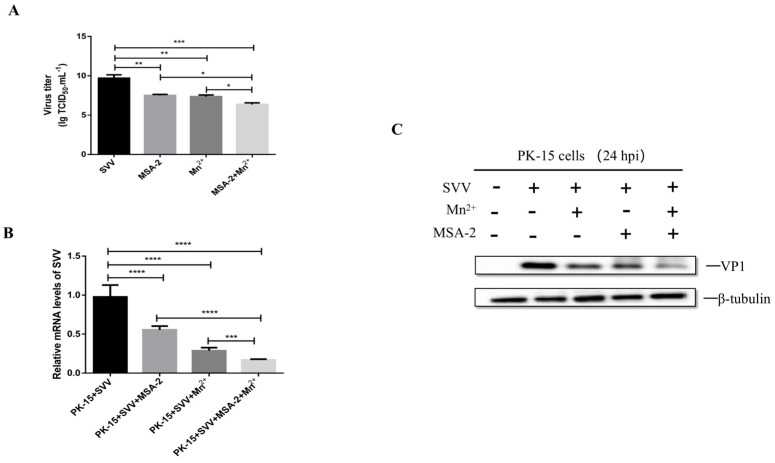
The combination of MSA-2 and Mn^2+^ can better inhibit SVV replication in PK-15 cells. PK-15 cells were treated with both 30 μM MSA-2 and 30 μM Mn^2+^ 1 h after SVV infection. Then, 24 h after virus infection, virus titer (**A**), virus RNA level (**B**) and VP1 protein expression (**C**) were detected by TCID_50_, RT-qPCR and Western blot. *, *p* < 0.05; **, *p* < 0.01; ***, *p* < 0.001; ****, *p* < 0.0001.

The studies above demonstrated that both MSA-2 and Mn^2+^ have potent inhibitory on SVV replication in the cells. In order to explore whether the MSA-2 combined with Mn^2+^ can induce a better inhibitory effect on SVV replication, the PK-15 cells were treated with 30 μM MSA-2 and 30 μM Mn^2+^, respectively, or co-treated with both 30 μM MSA-2 and 30 μM Mn^2+^ at 1 h after SVV infection. The virus titer, viral RNA level and VP1 protein expression were detected by TCID_50_ assay, RT-qPCR and Western Blot, respectively. The results showed that viral titers (Figure 7A), RNA levels (Figure 7B), and VP1 protein expression (Figure 7C) in the cells treated with both MSA-2 and Mn^2+^ were significantly lower than those in cells treated with either MSA-2 or Mn^2+^ alone, confirming that MSA-2 combined with Mn^2+^ enhanced inhibitory effect on SVV replication.

### 3.8. MSA-2 Synergized with Mn^2+^ in Activating STING

**Figure 8:**
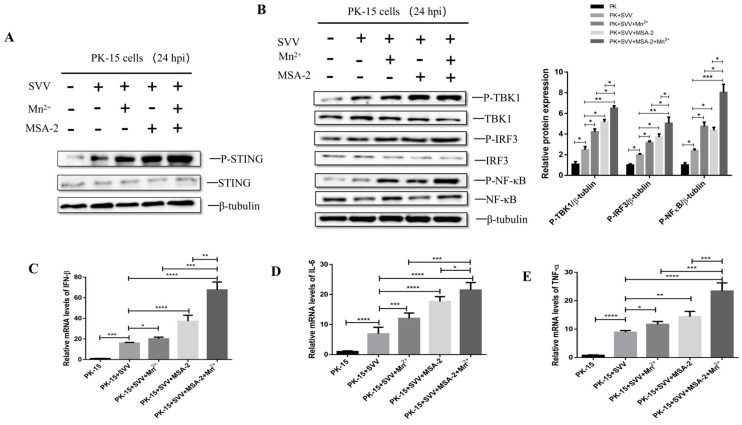
The combination of MSA-2 and Mn^2+^ can better activate innate immunity and induce cytokine expression. PK-15 cells were treated with both 30 μM MSA-2 and 30 μM Mn^2+^ 1 h after infection. P-STING protein expression was detected by Western blot (**A**), and P-TBK1, P-IRF3, and P-NFκB protein levels were detected by Western blot (**B**). PK-15 cells were treated with both 30 μM MSA-2 and 30 μM Mn^2+^ 1 h after infection, and the mRNA levels of IFN-β (**C**), IL-6 (**D**), and TNF-α (**E**) were detected by RT-qPCR 24 h after virus infection. *, *p* < 0.05; **, *p* < 0.01; ***, *p* < 0.001; ****, *p* < 0.0001.

In order to explore whether MSA-2 and Mn^2+^ have a synergistic effect in activating the STING signal pathway, the PK-15 cells were treated with 30 μM MSA-2 and 30 μM Mn^2+^, respectively, or co-treated with 30 μM MSA-2 and 30 μM Mn^2+^ at 1 h after SVV infection. At 24 hpi, the levels of P-STING and its related proteins in the downstream signal pathway were detected by Western blot. The results showed that the MSA-2 combined with Mn^2+^ could promote the phosphorylation of STING, TBK1, IRF3 and NFκB, and the levels of their phosphorylation were significantly higher than those in the cells treated with MSA-2 or Mn^2+^, respectively (Figure 8A,B). In addition, the mRNA levels of IFN-β (Figure 8C), IL-6 (Figure 8D) and TNF-α (Figure 8E) in SVV-infected cells co-treated with MSA-2 and Mn^2+^ were significantly higher than those in the cells only treated with either MSA-2 or Mn^2+^.

### 3.9. MSA-2 Combined with Mn^2+^ in Enhancing Anti-Virus Activity Depends on Activation of STING

**Figure 9:**
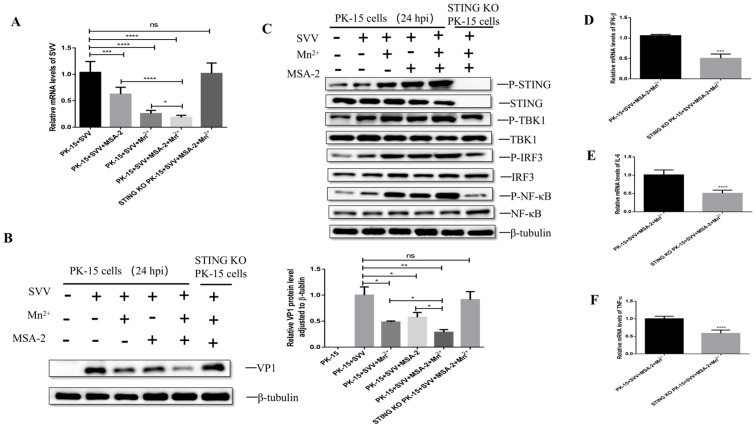
The synergism of MSA-2 and Mn^2+^ depends on STING. PK-15 cells or STING knockout PK-15 cells were treated with both 30 μM MSA-2 and 30 μM Mn^2+^ 1 h after infection. Viral RNA level (**A**) and SVV VP1 proteins (**B**) were detected by RT-qCPR and Western blot 24 h after infection. (**C**) Cells were treated with both 30 μM MSA-2 and 30 μM Mn^2+^ 1 h after infection, and P-TBK1, P-IRF3, and P-NFκB protein levels were detected by Western blot 24 h after infection. Cells were treated with both 30 μM MSA-2 and 30 μM Mn^2+^ 1 h after infection, and the mRNA levels of IFN-β (**D**), IL-6 (**E**), and TNF-α (**F**) were detected by RT-qPCR 24 h after virus infection.*, *p* < 0.05; **, *p* < 0.01; ***, *p* < 0.001; ****, *p* < 0.0001.

In order to explore whether the synergistic effect of MSA-2 and Mn^2+^ depends on the activation of STING, the PK-15 cells or STING knockout PK-15 cells were co-treated with 30 μM MSA-2 and 30 μM Mn^2+^ at 1 h after infection. The levels of viral RNA and VP1 protein in the cells were detected by RT-qPCR and Western blot at 24 hpi. The results showed that compared with wild-type cells group, the synergistic inhibitory effect of MSA-2 and Mn^2+^ on viral RNA and VP1 protein expression was decreased in the STING knockout cells (Figure 9A,B). Further investigation showed that that compared with SVV-infected PK-15 cells co-treated with MSA-2 and Mn^2+^, the levels of P-TBK1, P-IRF3, and P-NFκB (Figure 9C) also decreased in the STING knockout cells, indicating that the synergistic inhibitory effect of MSA-2 and Mn^2+^ depends on the activation of STING. Moreover, the levels of IFN-β, IL-6 and TNF-α mRNA were also down-regulated after STING knockout (Figure 9D–F). These results suggest that STING is necessary for the synergistic antiviral effect of MSA-2 and Mn^2+^.

## 4. Discussion

It has been demonstrated that MSA-2 can activate mouse and human STING genes, induce the phosphorylation of TBK1 and IRF-3 and activate the STING signal pathway in the human leukemia monocytic cell line (THP-1 cells) and induce the expression of IFN-β, IL-1β and TNF-α via the action of TBK1 [4,15]. In addition, existing studies have proved that MSA-2 has good anti-tumor activity [4,16]. In our study, we have demonstrated that as in human and mouse cells [4], MSA-2 can be used as a STING agonist in porcine cells to induce STING phosphorylation and has inhibitory effect on the viral replication in vitro in a dose-dependent manner.

As a transition metal, manganese exists in a variety of oxidation states, of which Mn^2+^ is one of the most common forms in biological species [17]. Manganese is an important component of many kinds of metalloenzymes and can exert its functions by regulating various manganese-dependent enzymes, and it is an essential trace element for various physiological processes [6,7,18]. It was recently found that Mn^2+^ was released from membrane-sealed organelles and accumulated in the cytoplasm in the cells infected with DNA virus. Such Mn^2+^ not only can combine with the DNA sensor cGAS to increase the recognition ability of cGAS to dsDNA, but it also can enhance the binding affinity of cGAMP and STING, thus increasing the activity of STING [8]. In addition, Manganese deficiency in mice showed reduced cytokine production and increased susceptibility to DNA virus infection [8]. Therefore, Mn^2+^ can be used as a cGAS-STING activator to promote the phosphorylation of STING to induce the expression of IFN-β and cytokines, leading to anti-DNA virus infection. [19,20]. However, whether the Mn^2+^ has anti-RNA virus effects is still unclear. In our study, it was found that Mn^2+^could inhibit the replication of SVV in PK-15 cells in a dose-dependent manner, regardless of whether before or after virus infection. It is unclear if the MSA-2 and Mn^2+^ could have impacts on the spreading virus infection in the cells. We found that MSA-2 or Mn^2+^ could activate STING and then promote the phosphorylation of TBK1, IRF3, and NFκB to induce the expression of type I IFN (IFN-β) as well as IL-6 and TNF-α mRNA in PK-15 cells infected with SVV. When STING was knocked out, the antiviral effects of MSA-2 and Mn^2+^ were weakened, indicating both MSA-2 or Mn^2+^ induce a STING-dependent anti-virus immune response in the cells. We also found that IFN-β was still detectable even though its mRNA level was reduced in STING-KO cells upon virus infection. SVV is an RNA virus which is recognized by retinoic acid-inducible gene I (RIG-I) in the cells, leading to activating TBK1 and NF-κB to induce cytokine production [21]. Furthermore, it was reported that MSA-2 or Mn^2+^can induce the phosphorylation of TBK1 and IRF-3 in the human cells [8,10,15], so it could be interesting to investigate if MSA-2 or Mn^2+^ in addition to the direct activation of STING could also activate TBK1 and IRF-3 of STING downstream to induce IFN-β expression in the porcine cells. A recent study showed that Mn^2+^ coordinated with CDN STING agonists to effectively deliver STING agonists to immune cells and initiate robust anti-tumor immunity [22]. In addition, Mn^2+^ could act together with MSA-2 to trigger downstream signaling by recruiting and activating TBK1 and IRF3 [23]. In our study, when we treated PK-15 cells with both MSA-2 and Mn^2+^, we found that in comparison with the use of MSA-2 or Mn^2+^ separately, the combination of MSA-2 and Mn^2+^ could have a better inhibitory effect on the replication of SVV in PK-15 cells. In addition, the phosphorylation levels of STING, TBK1, IRF3 and NFκB were significantly higher, and the expressions of IFN-β, IL-6 and TNF-α were also increased. This result confirmed that the combination of MSA-2 and Mn^2+^ has a synergistic anti-RNA virus effect. But further research is required to confirm whether MSA-2 and Mn^2+^ have broad-spectrum synergistic antiviral effects in vitro and in vivo. In addition, we also found that the antiviral effect of MSA-2 or Mn^2+^ has a more significant impact on the viral replication in the cells treated with MSA-2 or Mn^2+^ after infection. A recent study demonstrated that the combined regimen of Mn^2+^ and anti-PD-1 antibody showed promising efficacy, exhibiting type I IFN induction, manageable safety and revived responses to immunotherapy in most patients with tumors [11]. Our recent study showed that Mn^2+^ can be highly effective in protecting C57BL/6N mice from being infected with FMDV [9], indicating that Mn^2+^ is an effective antiviral additive for controlling viral infection in vivo. Therefore, it would be beneficial to carry out further study to explore their therapeutic potential against viral infection.

In conclusion, the present study has proved that both MSA-2 and Mn^2+^ can inhibit SVV replication in vitro, and both of them can activate downstream signal pathways by activating STING. More importantly, MSA-2 combined with Mn^2+^ has synergistic effects on antiviral activity in vitro, and further study on their antiviral effects is required through animal experiments.

## Figures and Tables

**Table 1:** Primer sequences used in our study.

Primers Name	Primers Sequence (5′–3′)
SVV	qF: GTGGGAAGGTATCTTTCGTG qR: TCATAGTGGTGAGACTTTGGGC
GAPDH	qF: GTCGGTTGTGGATCTGACCT qR: AGCTTGACGAAGTGGTCGTT
IFN-β	qF: TGCAACCACCACAATTCCAGAAGG qR: TGACGGTTTCATTCCAGCCAGTG
IL-6	qF: GCTGCTTCTGGTGATGGCTACTG qR: AGAGCATTTTGTCTGAGGTGGCATC
TNF-α	qF: CCTCATCTACTCCCAGGTCCTCTTC qR: GATGCGGCTGATGGTGTGAGTG

## Data Availability

The data that support the findings of this study are available from the corresponding author upon reasonable request.

## References

[1] Webb L.G., Fernandez-Sesma A. (2022). RNA viruses and the cGAS-STING pathway: Reframing our understanding of innate immune sensing. Curr. Opin. Virol..

[2] Li M., Ferretti M., Ying B., Descamps H., Lee E., Dittmar M., Lee J.S., Whig K., Kamalia B., Dohnalová L. (2021). Pharmacological activation of STING blocks SARS-CoV-2 infection. Sci. Immunol..

[3] Humphries F., Shmuel-Galia L., Jiang Z., Wilson R., Landis P., Ng S.-L., Parsi K.M., Maehr R., Cruz J., Morales A. (2021). A diamidobenzimidazole STING agonist protects against SARS-CoV-2 infection. Sci. Immunol..

[4] Pan B.-S., Perera S.A., Piesvaux J.A., Presland J.P., Schroeder G.K., Cumming J.N., Trotter B.W., Altman M.D., Buevich A.V., Cash B. (2020). An orally available non-nucleotide STING agonist with antitumor activity. Science.

[5] Yi M., Niu M., Wu Y., Ge H., Jiao D., Zhu S., Zhang J., Yan Y., Zhou P., Chu Q. (2022). Combination of oral STING agonist MSA-2 and anti-TGF-β/PD-L1 bispecific antibody YM101: A novel immune cocktail therapy for non-inflamed tumors. J. Hematol. Oncol..

[6] Horning K.J., Caito S.W., Tipps K.G., Bowman A.B., Aschner M. (2015). Manganese Is Essential for Neuronal Health. Annu. Rev. Nutr..

[7] Kwakye G.F., Paoliello M.M.B., Mukhopadhyay S., Bowman A.B., Aschner M. (2015). Manganese-Induced Parkinsonism and Parkinson’s Disease: Shared and Distinguishable Features. Int. J. Environ. Res. Public Health.

[8] Wang C., Guan Y., Lv M., Zhang R., Guo Z., Wei X., Du X., Yang J., Li T., Wan Y. (2018). Manganese Increases the Sensitivity of the cGAS-STING Pathway for Double-Stranded DNA and Is Required for the Host Defense against DNA Viruses. Immunity.

[9] Zhang Z., Zhang R., Yin J., Zhao S., Qin X., Chen F., Yang Y., Bai L., Guo Z., Wu Y. (2023). Antiviral Effect of Manganese against Foot-and-Mouth Disease Virus Both in PK15 Cells and Mice. Viruses.

[10] Sui H., Chen Q., Yang J., Srirattanapirom S., Imamichi T. (2022). Manganese enhances DNA or RNA-mediated innate immune response by inducing phosphorylation of TANK-binding kinase 1. iScience.

[11] Lv M., Chen M., Zhang R., Zhang W., Wang C., Zhang Y., Wei X., Guan Y., Liu J., Feng K. (2020). Manganese is critical for antitumor immune responses via cGAS-STING and improves the efficacy of clinical immunotherapy. Cell Res..

[12] Hales L.M., Knowles N.J., Reddy P.S., Xu L., Hay C., Hallenbeck P.L. (2008). Complete genome sequence analysis of Seneca Valley virus-001, a novel oncolytic picornavirus. J. Gen. Virol..

[13] Joshi L.R., Fernandes M.H.V., Clement T., Lawson S., Pillatzki A., Resende T.P., Vannucci F.A., Kutish G.F., Nelson E.A., Diel D. (2016). Pathogenesis of Senecavirus A infection in finishing pigs. J. Gen. Virol..

[14] Liu J., Ren X., Li Z., Xu G., Lu R., Zhang K., Ning Z. (2018). Genetic and phylogenetic analysis of reemerged novel Seneca Valley virus strains in Guangdong Province, 2017. Transbound. Emerg. Dis..

[15] Kabelitz D., Zarobkiewicz M., Heib M., Serrano R., Kunz M., Chitadze G., Adam D., Peters C. (2022). Signal strength of STING activation determines cytokine plasticity and cell death in human monocytes. Sci. Rep..

[16] Yu J., He S., Zhang C., Xu C., Huang J., Xu M., Pu K. (2023). Polymeric STING Pro-agonists for Tumor-Specific Sonodynamic Immunotherapy. Angew. Chem. Int. Ed. Engl..

[17] Takeda A. (2003). Manganese action in brain function. Brain Res. Rev..

[18] Waldron K.J., Rutherford J.C., Ford D., Robinson N.J. (2009). Metalloproteins and metal sensing. Nature.

[19] Sun S., Xu Y., Qiu M., Jiang S., Cao Q., Luo J., Zhang T., Chen N., Zheng W., Meurens F. (2023). Manganese Mediates Its Antiviral Functions in a cGAS-STING Pathway Independent Manner. Viruses.

[20] Zhang X., Liu J., Wang H. (2022). The cGAS-STING-autophagy pathway: Novel perspectives in neurotoxicity induced by manganese exposure. Environ. Pollut..

[21] Loo Y.M., Gale M. (2011). Immune signaling by RIG-I-like receptors. Immunity.

[22] Sun X., Zhang Y., Li J., Park K.S., Han K., Zhou X., Xu Y., Nam J., Xu J., Shi X. (2021). Amplifying STING activation by cyclic dinucleotide-manganese particles for local and systemic cancer metalloimmunotherapy. Nat. Nanotechnol..

[23] Lu Q., Chen R., Du S., Chen C., Pan Y., Luan X., Yang J., Zeng F., He B., Han X. (2022). Activation of the cGAS-STING pathway combined with CRISPR-Cas9 gene editing triggering long-term immunotherapy. Biomaterials.

